# Late Holocene environmental change and anthropogenic: Ecosystem interaction on the Laikipia Plateau, Kenya

**DOI:** 10.1007/s13280-021-01554-6

**Published:** 2021-06-16

**Authors:** Veronica Muiruri, Rob Marchant, Stephen M. Rucina, Louis Scott, Paul J. Lane

**Affiliations:** 1 Palynology & Palaeobotany Section. Department of Earth Sciences, National Museums of Kenya, Nairobi, Kenya; 2 Department of Environment and Geography, York Institute for Tropical Ecosystems, University of York, Heslington, York, North Yorkshire YO10 5NG UK; 3 Department of Plant Sciences, University of the Free State, Bloemfontein, South Africa; 4 Department of Archaeology, University of Cambridge, Downing Street, Cambridge, CB2 3DZ UK; 5 School of Geography, Archaeology and Environmental Studies, University of the Witwatersrand, Johannesburg, South Africa

**Keywords:** Charcoal, Fire history, Fungal spores, Land use management, Pollen, Rangelands

## Abstract

East African ecosystems have been shaped by long-term socio-ecological–environmental interactions. Although much previous work on human–environment interrelationships have emphasised the negative impacts of human interventions, a growing body of work shows that there have also often been strong beneficial connections between people and ecosystems, especially in savanna environments. However, limited information and understanding of past interactions between humans and ecosystems of periods longer than a century hampers effective management of contemporary environments. Here, we present a late Holocene study of pollen, fern spore, fungal spore, and charcoal analyses from radiocarbon-dated sediment sequences and assess this record against archaeological and historical data to describe socio-ecological changes on the Laikipia Plateau in Rift Valley Province, Kenya. The results suggest a landscape characterised by closed forests between 2268 years before present (cal year BP) and 1615 cal year BP when there was a significant change to a more open woodland/grassland mosaic that continues to prevail across the study area. Increased amounts of charcoal in the sediment are observed for this same period, becoming particularly common from around 900 cal year BP associated with fungal spores commonly linked to the presence of herbivores. It is likely these trends reflect changes in land use management as pastoral populations improved and extended pasture, using fire to eradicate disease-prone habitats. Implications for contemporary land use management are discussed in the light of these findings.

## Introduction

Understanding the detail of longer-term climate–human–ecosystem interaction is essential for the sound and sustainable management of cultural landscapes. Past research in eastern Africa has provided insights into the relationships between human activities, rainfall, soil fertility, wildlife, livestock grazing, and deforestation over both decadal and centennial scales, particularly with respect to pastoral communities and their links to conservation of rangelands and wildlife across Africa (e.g. Githumbi et al. 2018; Phelps et al. 2020; Sitters et al. 2020). Parallel studies have also explored, to good effect, the intersection of natural processes and anthropogenic factors, such as fuel wood use, agricultural intensification, and natural resources extraction in farming settings (e.g. Sulas et al. 2009; Lang and Stump 2017). Nonetheless, establishing this long-term historical context remains especially problematic for more lowland settings in eastern Africa, as the palaeoecological signatures for landscape transformation have been documented principally from mountain environments (Marchant et al. 2018), where suitable natural sedimentary archives, such as lake and bog deposits, can be found. Lake, swamp, and bog sediments trap evidence of the surrounding environments for palaeoenvironmental reconstruction, with pollen from surrounding vegetation forming a key component of this evidence base. Other proxies such as charcoal, grass cuticles, molecular markers, fungal spores, plankton, fossil insects, diatoms, and ostracods have all provided useful supplementary paleoenvironmental and climatic insights (Marchant et al. 2018). Palaeoenvironmental studies also provide the evidence base of past land use transitions and are fundamental for assessing the consequences of various anthropogenic drivers of change inferred from archaeological and historical data (Gillson and Marchant 2014).

Despite considerable and growing interest in the interrelationship between human activities and environmental change, equifinality makes palaeoenvironmental signals difficult to separate. Documenting the long-term history of climate–human–landscape interaction in East Africa, for example, is complicated by the challenge of discriminating between the palaeoecological signatures of human activities and the response of local vegetation ecotones (van der Plas et al. 2019). Climate change events (for example a shift to a drier climate) may produce the same signal, such as an increase in grasses, as a shift to increased pastoral activity. These problems are exacerbated as people practicing nomadic or transhumant lifestyles often exert a relatively light footprint on the region’s savanna environments, making the detection of former habitation sites and other activity areas particularly challenging (Shahack-Gross et al. 2003). By applying a multiproxy approach, and combining archaeological and palaeoecological investigations from the same context, it is possible to separate out these effects, and indicate what ecological legacies cultures imparted on their environment, and how these altered during periods of land use change (Robertshaw et al. 2004). However, such cross-disciplinary research is often carried out in an overly simplistic manner that can lead to a misinterpretation of the results and overly deterministic perspectives (Coombes and Barber 2005). Previous research has demonstrated the importance of past environmental evidence for contextualising archaeological findings, particularly where there are strong environmental impacts such as those following the introduction of iron working, changes in agriculture, transition to nucleated settlement patterns, and/or the arrival of new populations with different subsistence strategies (Taylor et al. 1999; Iles et al. 2018). There is still a pressing need, nonetheless, to bridge the divide that exists between palaeoenvironmental and archaeological evidence (Arponen et al. 2019).

With that goal in mind, this study documents historical changes in a savanna ecosystem and, through applying an interpretive lens from local archaeological sites, aims to determine the role of natural and anthropogenic processes on the Laikipia Plateau in Rift Valley Province, Kenya. We present pollen, fern spore, fungal spore, and charcoal analyses derived from radiocarbon-dated sediment sequences to describe ecosystem changes that have taken place in this study area over the late Holocene. As the stratigraphic records were recovered from an area in proximity to archaeological sites, this allows us to avoid pitfalls commonly associated with research that correlates archaeological and palaeoecological results across different environmental settings. The research focuses on the interactions between ‘human’ and ‘natural’ factors, particularly the impact of climate, agriculture, and subsistence pastoralism on local ecosystems. In addition to detailing the environmental backdrop to the in situ archaeology, results from this research are directly relevant to understanding ongoing ecosystem and land use change on the Laikipia Plateau. This is an area of increasing and rapid transition that has prompted multiple debates about the use of land for conservation, group ranches, pastoral rangelands, and agricultural production (Roden et al. 2016; Pas Schrijver 2019). The results also provide an insight on the pre-modern environment and patterns of resource management that could potentially inform future efforts at landscape and habitat restoration.

## Study area

**Fig. 1:**
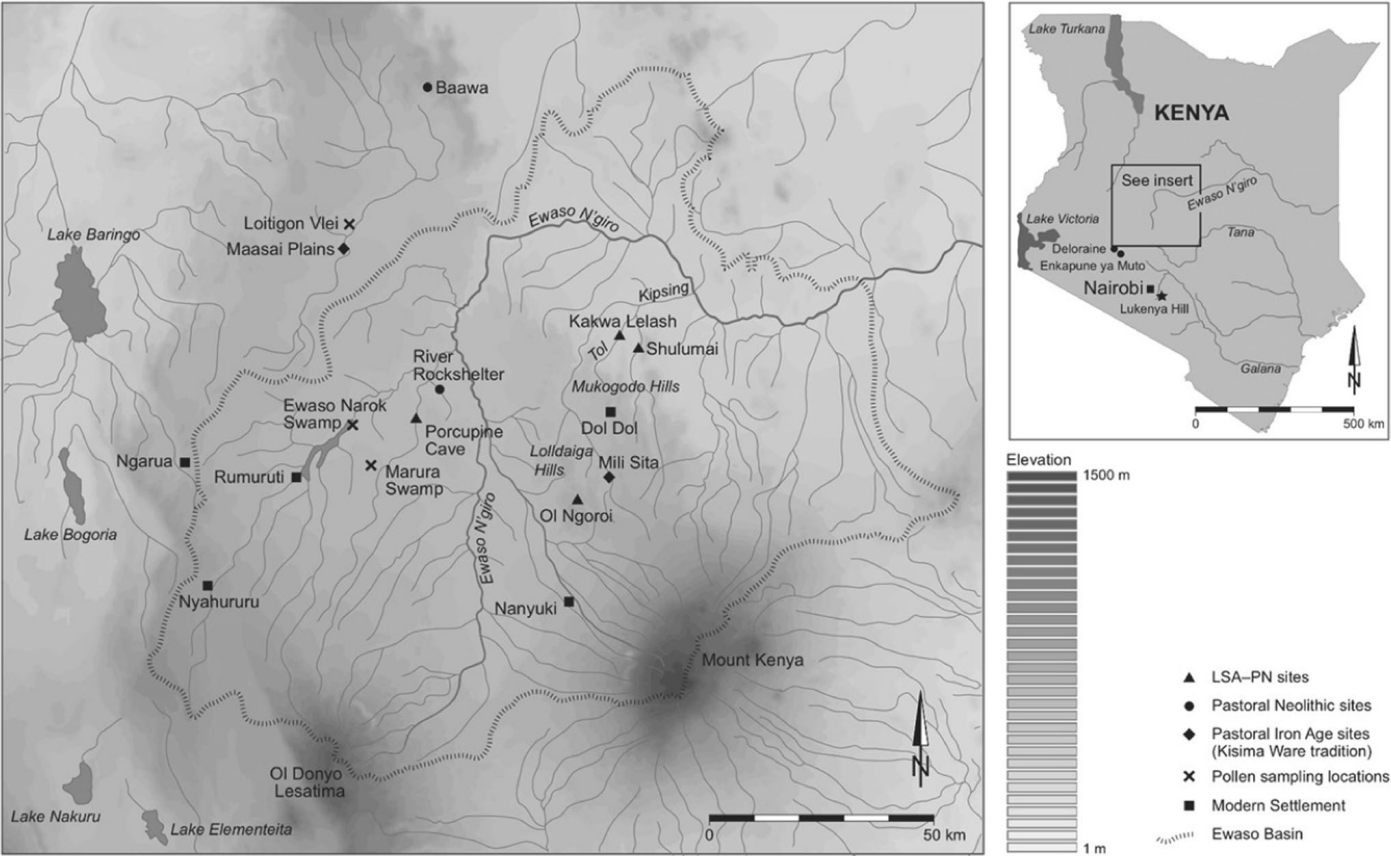
Map showing the location of the Marura coring site on the Laikipia Plateau, central Kenya, in relation to other locations and key archaeological sites mentioned in the text (prepared by Tim Evans)

**Fig. 2:**
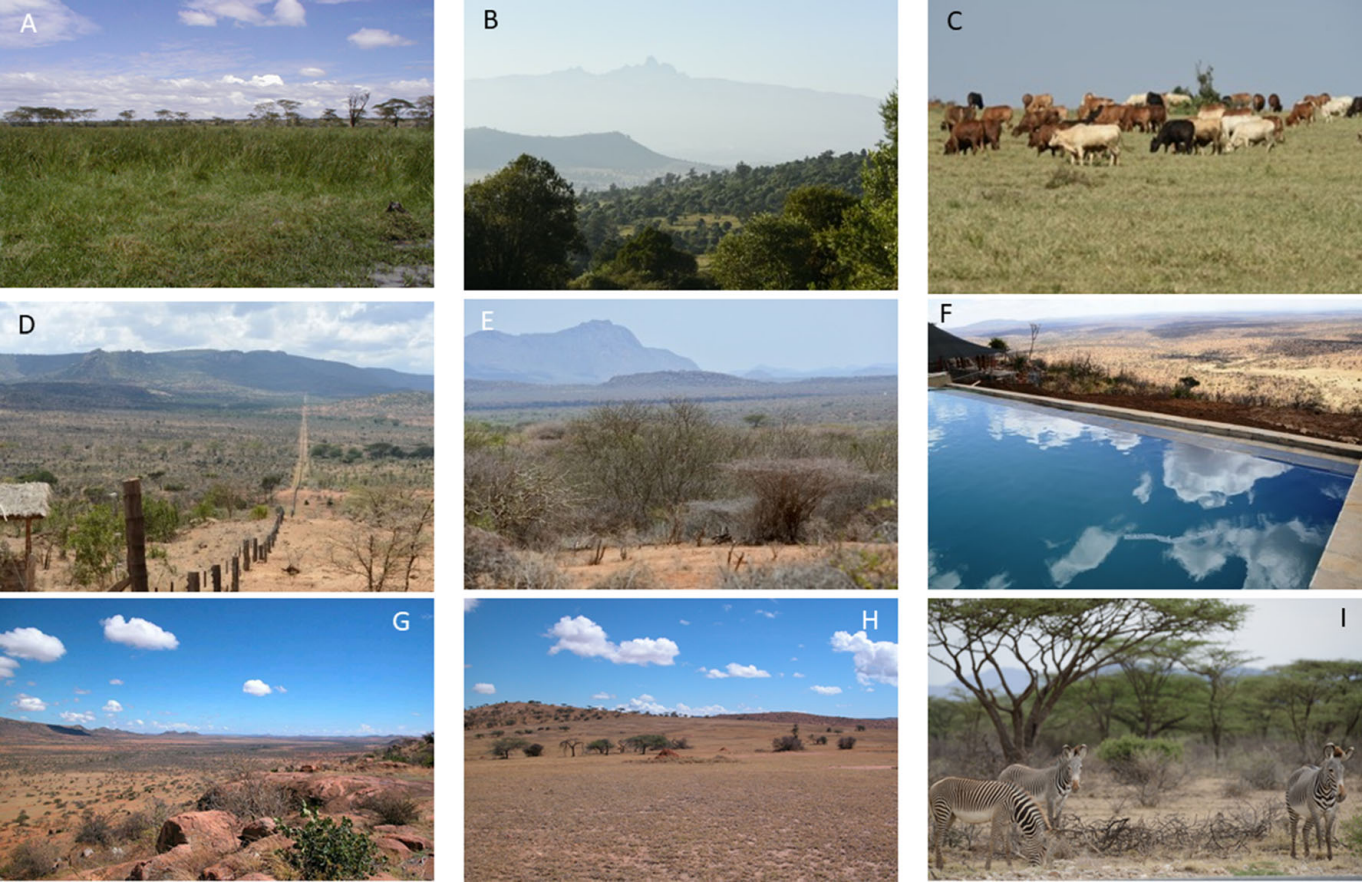
Photomontage showing some of the contemporary issues and scenes from the Laikipia Plateau. Marura Swamp is where the sediment core was collected to provide the palaeoenvironmental data presented here (**a**); view from the southern part of the Laikipia Plateau towards Mount Kenya (**b**); one of the mainstays of land use on the Laikipia Plateau are cattle, kept either by semi-nomadic pastoral communities or within large commercial livestock ranches (**c**); since the colonial period the subdivision and fencing of the Laikipia Plateau has been an ongoing and continuing issue (**d**), the settlement of the Laikipia Plateau has been accompanied by a series of invasive plants such as Opuntia and *Acacia reficiens* that degrade the grazing resource (**e**); agricultural and pastoral land uses are often adjacent to tourism operations, these often quite exclusive high-end private conservancies (**f**); one of the private livestock ranches is Lolldiaga (**g**) that contains a series of archaeological sites such as Maili Sita (**h**); one of the many endemic animals of high conservation concern is the Grevy’s Zebra (**i**) (Photo Credits: Rob Marchant **a**–**f**, **i**; Paul Lane **g**–**h**)

The research focuses on a new palaeoecological record from Marura swamp (Muiruri 2008), located within the savanna forest ecotone on Laikipia Plateau, Kenya (Fig. 1). Laikipia is bordered by the highlands of Mt. Kenya and the Nyandarua (formerly Aberdare) Range in the south and west, respectively, while to the north and east it merges gradually into extensive plains extending to Lake Turkana. The Laikipia Plateau is characterised by considerable environmental variation due to rain shadow effects and topographic diversity (Fig. 2). For example, the eastern flanks of Mt. Kenya receive annual precipitation of approximately 2000 mm yr^−1^, whereas the western parts receive 500–700 mm yr^−1^ and the central and northern parts of the plateau only 300–500 mm yr^−1^. Rain falls in two seasons, with 80% falling around March–May and October–November. Rainfall is increasingly unpredictable and drought years are frequent (Gichuki et al. 1998).

The present vegetation on the central plateau comprises dry savanna dominated by *Acacia* and *Themeda* species; this gives way to thorn savanna towards the north with *Acacia drepanolobium*, *A. tortilis*, and *Croton dichogamus* common (Fig. 2). Across much of the Laikipia Plateau, probably because of heavy grazing by both wild and domestic animals, the undergrowth is locally very sparse (Fig. 2). Without continued grazing and clearing by people through fire, large areas would revert to closed bush thicket, an increasing phenomenon under continued and ongoing expansion of woody plants across African savanna (Bond and Midgley 2012). Today, land use comprises a mix of wildlife conservancies, large farms, ranches, smallholder market gardening and subsistence farming, and transhumant livestock herding by pastoral communities (Pas Schrijver 2019) (Fig. 2). These contrasting land uses sometimes result in conflict as mobile pastoral populations and migratory wildlife compete for space and grazing resources within an increasingly populated, divided, and fenced landscape (Gravesen 2020).

The natural vegetation surrounding the sampling site is dominated by *Cyperus* with *Acacia drepanolobium* and *Euclea divinorum*. Marura swamp is surrounded by settlements and irrigated cultivation dominated by Brassicaceae, *Casuarina, Eucalyptus, Grevillea robusta,* and *Zea mays*. Cabbage, spinach, and other vegetables are grown during the dry season through irrigation and form an important source of local income. The wetland is important for the bordering communities and their animals after the Ol’ Pejeta ranch was fenced to prevent encroachment. The swamp provides habitat for harvestable plants and is a vital source of drinking water for stock, especially when short rains between March and April fail and during longer droughts. Between the 1920s and the 1970s, the dominant land use was large-scale ranching and nomadic pastoralism with pockets of smallholder agriculture especially after land redistribution following independence in 1963 (Kohler 1987). More recently, there has been a strong trend for previously nomadic communities to settle along riverine and around wetland areas, at the same time as pre-existing permanent settlements have expanded.

## Materials and methods

**Fig. 3:**
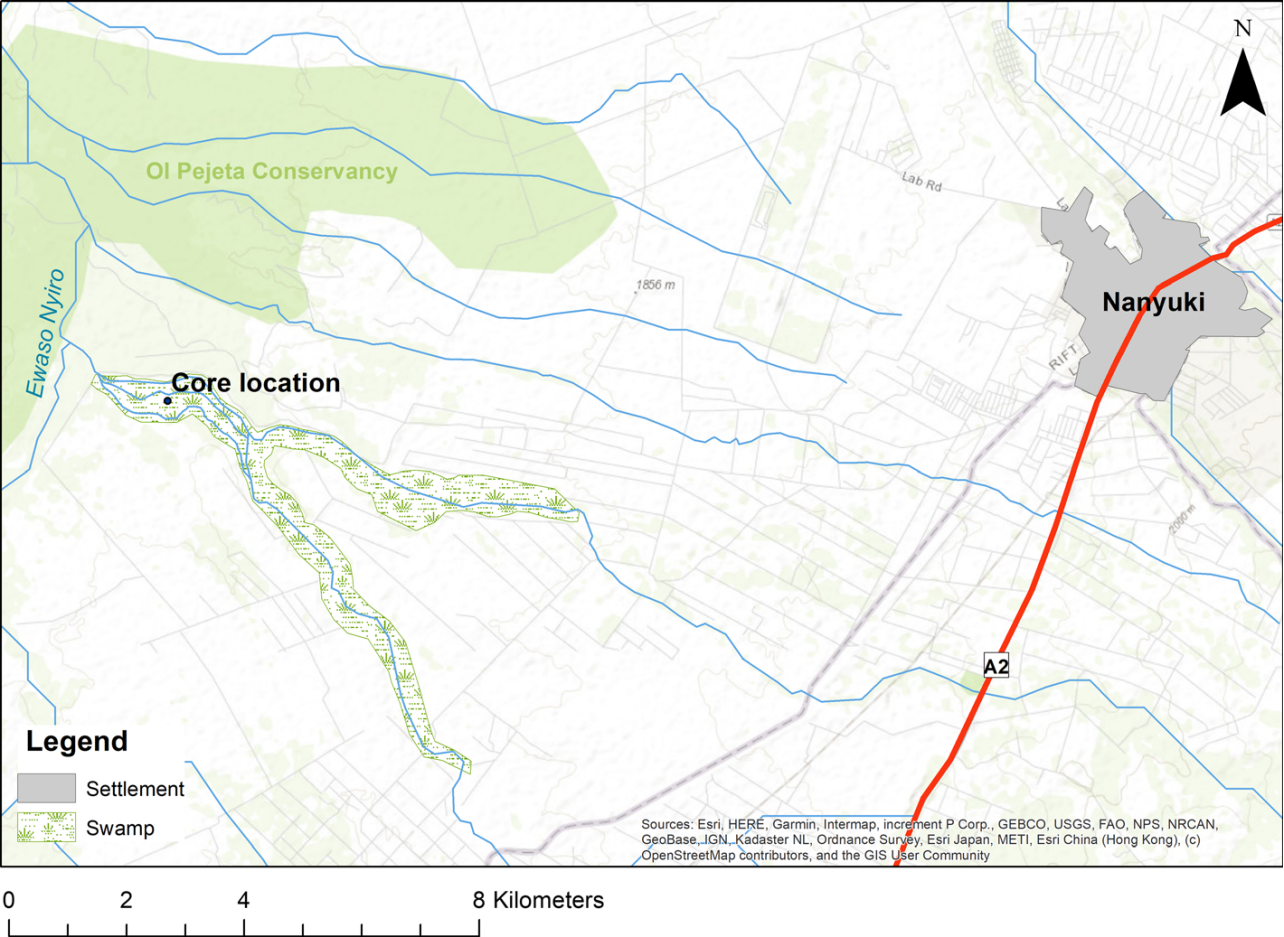
Location and extent of Marura Swamp and its associated drainage network. The approximate location of the coring site is marked by a dot (prepared by Stefania Merlo)

Two sediment cores were collected for this research in September 2006: one from Marura Swamp and one from Ewaso Narok Swamp near Rumuruti (Muiruri 2008) using a 50-cm-long, 5-cm-diameter Russian D-section corer from adjacent boreholes. Due to dating challenges with the Ewaso Narok core, we only present the sedimentary record from Marura Swamp (Fig. 3). Marura Swamp (centred on 0° 0′ 2.11″ S 36° 55′ 58.33″ E) is about 2 km long, although it fluctuates in extent during the dry and wet seasons as the level of the Mutara River changes. The sediment core was collected near the centre of Marura Swamp at an altitude of 1800 m. Sediments were found to be relatively variable in composition in the upper part (c. 0–94 cm) of the core, and more homogenous at greater depths. Dark, fibrous peat, grey-brown and organic-rich clays, interspersed with rootlets, characterised the upper and middle section (c. 0–175 cm) of the sediment core. Brown-grey clays with varying amounts of sand comprised sediments from c. 175 to 250 cm depth. Hard, more compact dark grey clays with a few distinct black laminations were characteristic of all deeper sediments recovered from 250 to 400 cm, the core base.

### Radiocarbon dating and sediment analysis

Accelerator mass spectrometry (AMS) radiocarbon dating of five bulk sediment subsamples provided a geochronology for the Marura sediment core. Samples were submitted to either Beta Direct AMS or Beta Analytic, USA, and were alkali-acid–alkali pre-treated, combusted to CO_2_ and reduced to graphite on a catalyst for AMS. Measured ^14^C ages were corrected for isotopic fractionation with δ^13^C values by the laboratory service provider. Samples were chosen to provide age constraints on periods of vegetation change or to provide rangefinder dates to enhance the chronological control.

Sediments were prepared for microfossil pollen and fungal spores based on modified standard procedures published in Faegri and Iversen (1989) and Van Geel and Anderson (1988), respectively. The modification comprised use of chemical treatment involving KOH, acetolysis mixture (Erdtman 1960), and HF to remove humic acids, cellulose, and silica (Faegri and Iversen 1964). The pollen concentrate was mounted in glycerine jelly. A total of 500 pollen grains were counted for each sample if possible and identification carried out to the lowest taxonomic level based on the reference collection at the National Museums of Kenya.

Microscopic charcoal was counted on slides prepared for pollen analysis. Charcoal is regarded as black, uniformly opaque, angular particles with a long axis greater than 2.9 µm. Charcoal occurrence at 80 depths were counted using the point counting method adapted from Clark (1982) to estimate the area of charcoal on each slide. The technique involved counting charcoal particles under a magnification of x400 along transects spaced 2 mm apart on the slide. Size classing of charcoal fragments was carried out, following an adaptation of the method devised by Waddington (1969), by placing fragments into size-classes depending on their estimated surface area. For this study, the length of the longest axis was used and every fragment of charcoal encountered along the transects was recorded in one of the three size-classes: 2.9–29 µm, 29–63 µm, and > 63 µm based on a calibrated graticule. The size limit of the lower and upper size-classes was set so as to exclude very small fragments (< 2.9 µm), and incorporate the few large particles that had passed through a 150 µm sieve.

## Results

### Chronology and age–depth curve for Marura

**Fig. 4:**
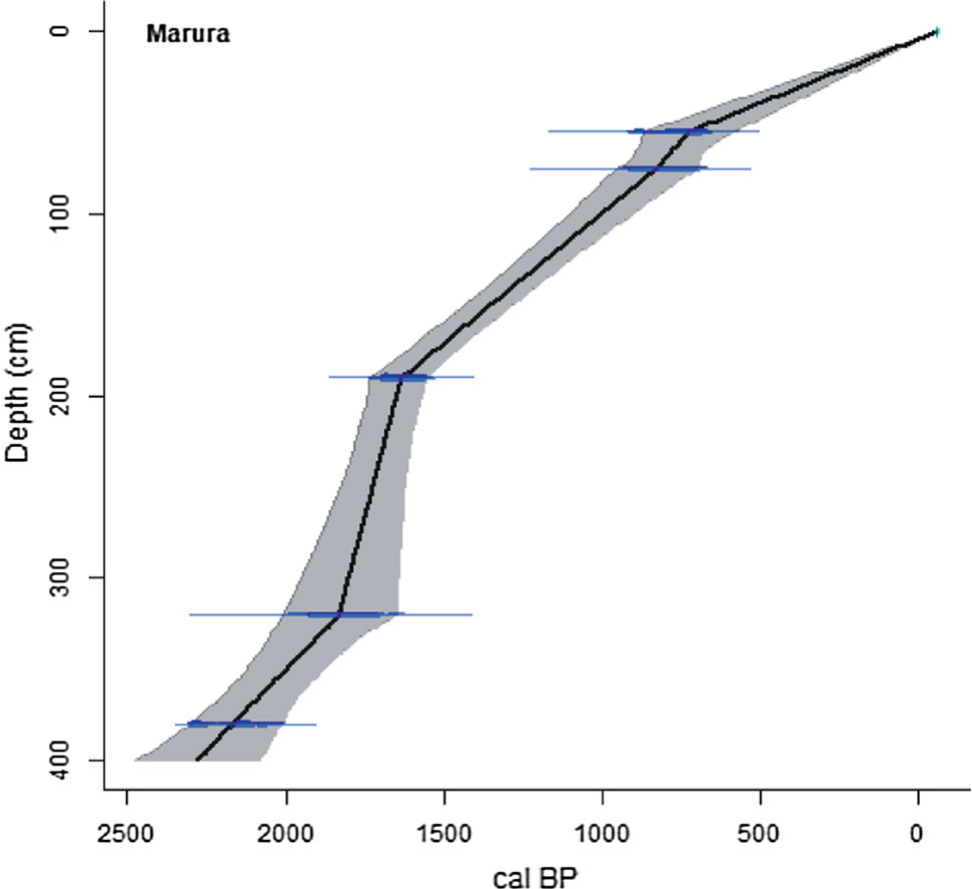
Marura age–depth curve. A linear relationship between age and depth is assumed between contiguous AMS ^14^C dates

The 400-cm-long sediment core consists mainly of organic-rich sediments with sand increasingly present towards the bottom of the core. In Table 1, the radiocarbon ages (^14^C year BP) were calibrated using the SHCal20 calibration curve (Hogg et al. 2020) and are expressed as calibrated years before present (cal year BP) relative to the Common Era calendar (CE 1950). Radiocarbon ages were not rounded prior to analysis (compare Stuiver and Pollach 1977). In Fig. 4, the radiocarbon dates and the top of the core (collected in 2006) were used to develop an age–depth model using linear interpolation between calibrated radiocarbon dates in the R statistical programming language version 4.0.0 using Clam 2.2 with the calibration curve SHCal20 (Blaauw 2010; Hogg et al. 2020; R Development Core Team 2020). The results of the AMS ^14^C dating of samples from Marura (Table 1) are stratigraphically consistent from the base at 2150 ± 40 cal year BP (380 cm) to the core top (Fig. 4). Based on the lithology and consistent age–depth profile, there is no indication for sedimentary hiatus although the sedimentation rate was variable.

**Table 1:** AMS radiocarbon dates and 13C/12C ratios (d13C ‰) values for Marura core

Sediment sample No.	Lab. no.	Depth (cm)	Conventional ^14^C age (yr BP) ± 1δ	Median Cal. ^14^C age	^13^C/^12^C δ^13^C (‰) value	Material dated
Marura 55	D-AMS 034916	55	812 ± 90	705 BP	0.31	Organic sediment
Marura 75	D-AMS 034917	75	879 ± 89	762 BP	0.31	Organic sediment
Marura 190	Beta 230859	190	1720 ± 40	1538 BP	− 19.1	Organic sediment
Marura 320	D-AMS 034918	320	1881 ± 79	1771 BP	0.27	Organic sediment
Marura 380	Beta 230859	380	2150 ± 40	2077 BP	− 19.1	Organic sediment

The AMS dates were calibrated using REV8.2 and calibration curve SHCal20 and presented as 2-sigma median calibrated age BP at 95% probability

#### Pollen and spore stratigraphy

**Fig. 5:**
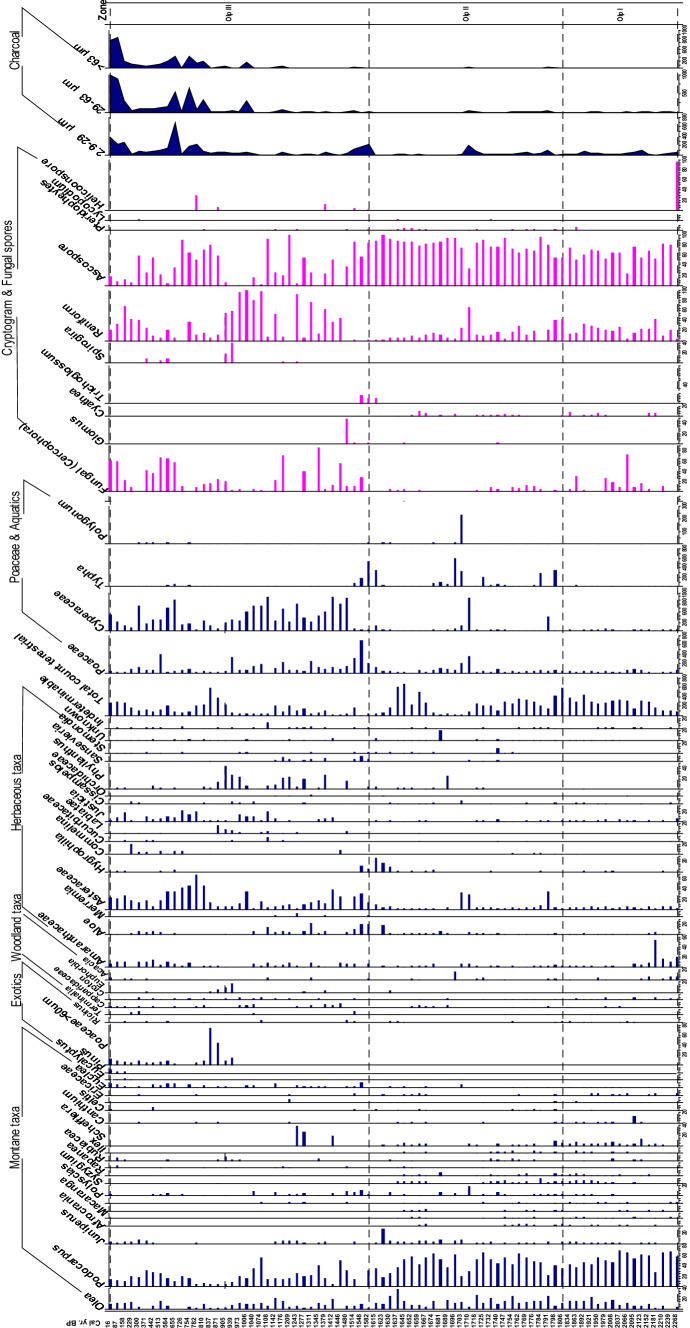
Marura pollen diagram showing down core percentages in changes of the selected taxa in broad ecological groupings of Afromontane, Woodland, Herbaceous, Poaceae, Charcoal and Fungal spores

Eighty samples from the Marura Swamp core were analysed for pollen, fern spore, fungal spore, and charcoal content. Ninety-three pollen and spore types were recognised to genus and family level. All but 28 samples were rich in well-preserved pollen fern spores and fungal spores (Fig. 5) with the total count (excluding damaged grains) ranging from 501 to 1246 pollen fern and fungal spores. By comparison, the recovery of pollen and spores from a number of levels (25 cm, 35 cm, 50–60 cm, 90–115 cm, 125 cm, 145–170 cm, 180–195 cm, 230–255 cm, and 400 cm) was poor, with pollen counts of less than 100 grains per sample. Samples were grouped according to their nonlocal pollen content, using stratigraphically constrained techniques of numerical clustering. The data were plotted using Tiliagraph Version 2.0.b.S (Grimm 1995). The boundaries of pollen assemblage zones were defined using a stratigraphically constrained clustering procedure (CONISS; Grimm 1987) with the Edwards and Cavalli-Sforza chord distances used as a dissimilarity measure. Pollen types that did not attain levels of greater than 2% in any sample were excluded from the clustering process, following the recommendations of Birks and Gordon (1985). Three pollen zones were recognised in the stratigraphically constrained zonation labelled Olp I, Olp II, and Olp III (Fig. 5). To aid in the interpretation, the pollen taxa are grouped into major ecological units of Afromontane, Woodland forest, Herbaceous, Grass and Aquatics and Exotic taxa (Fig. 5).

#### Zone Olp I (400–320 cm, 2268–1834 cal yr BP)

This zone is dominated by Afromontane taxa such as *Podocarpus* (25–80%), *Olea* (1–30%), *Juniperus* (0–5%), *Cyathea* (5–20%), *Polyscias* (0–5%), *Schefflera* (5–15%), and *Rapanea* (5%). The woodland forest category accounts for less than 20% of the pollen sum. Among the herbaceous taxa, Amaranthaceae contribute up to 60%, while Asteraceae and *Justicia* are rare in this zone recording less than 5%. Fungal (cercophora) spores dominate towards the upper boundary of this zone (0–98%) with Cyperaceae contributing up to 50% of the local pollen and cryptogam spores. The fungal spores are dominated by Acospore (20–80%), Reniform (10–40%) and *Valsaria type* (5–20%). The local pollen, fern spore, and fungal spore concentrations are generally low at the base of the zone, rising towards the upper pollen zone boundary (Fig. 5).

#### Zone Olp II (320–190 cm, 1806–1615 cal yr BP)

This zone is characterised by increased presence of Afromontane taxa as woodland taxa decrease. Apart from *Acacia,* which peaks to 20%, other woodland taxa (*Canthium*, *Dombeya*, *Croton, Euclea*, and Capparidaceae) range from 0 to 5% and are evenly distributed. Afromontane taxa such as *Juniperus* (1–20%), *Olea* (0–40%) and *Podocarpus* decreases, except for the uppermost sample, to the end of the zone (20–80%), with low occurrence of Ericaceae (0–10%), *Ilex* (0–10%), *Polyscias* (5–20%), *Rapanea* (0–15%), and *Syzygium* (0–15%). Among the herbaceous taxa, Poaceae are the most abundant taxa in the zone. *Aloe* (5–20%), Asteraceae (10–40%), *Artemisia* (< 5%), Amaranthaceae (1–10%), and *Sansevieria* (10%) are all present. Local fungal and fern spores are dominated by Acospore type (90%-40%), Reniform (5–60%), *Valsaria* type (0–20%), *Glomus* (0–40%), *Trichoglossum* (20%), *Spirogyra* (3%), *Cyathea*, *Lycopodium*, and *Helicoon* (0–15%). Local pollen was dominated by *Typha* (10–99%) with Cyperaceae having a high percentage (80%) at 52 cm (Fig. 5).

#### Zone Olp III (190–5 cm, 1582–16 cal yr BP)

Pollen zone Olp III extends from 190 cm to the core top. This pollen zone is characterised by a decrease of Afromontane taxa as woodland and herbaceous taxa rise. *Podocarpus* (60% to 5%), *Olea* (20–1%), *Juniperus* (5–0%), and *Polyscias* (10–0%) all decrease towards the core top. *Schefflera* reached a peak at 40%, before being absent from the record. *Alchornea, Myrica*, Nyctaginaceae, *Ricinus,* Rubiaceae, and *Terminalia* are present but low in percentages with the latter attaining 10% in the upper part of the core. *Canthium,* Capparidaceae, *Cleome, Commicarpus*, *Croton, Euclea*, and *Merremia* all attain less than 10%, with *Euphorbia* rising to 20%. Asteraceae are abundant ranging from 10 to 60% where other taxa are present in lower amounts: *Aloe* (0–10%), *Artemisia* (0–3%), Amaranthaceae (10–20%), Cucurbitaceae (0–5%), Orchidaceae (2%), *Rumex* (4%), and *Sansevieria* (1–5%). Cereal-like Poaceae grains measuring < 60 µm are increasingly common (10–80%) and a constant part of the pollen signature over the past 500 years following a large peak around 800–900 cal year BP. The exotic tree species of *Pinus* and *Eucalyptus* are consistently present in the uppermost five samples that provide a chronostratigraphic marker for the arrival of colonial forestry plantations. Other herbaceous taxa such as *Commelina* (10–20%), Labiatae (1–20%), *Justicia* (10–25%), *Phyllanthus* (1–45%), and *Solanum* (2–5%) are common. Cyperaceae dominates (40–80%) the local pollen and spores. Reniform spores and Ascospore type fluctuate widely (1–90%); indeed, fungal spores are well represented in this zone compared to other zones, ranging from 50 to 80%. Pteridophytes (5%), *Lycopodium* (3%), and *Helicoon* spores (5–20%) are all present (Fig. 5).

### Microscopic charcoal

All samples contained microscopic charcoal. There is a slight decline in the proportion of charcoal in the smallest size-class (2.9–29 µm) towards the core top, although it remains the most significant size-class (Fig. 5). Charcoal in the middle size-class (29–63 µm) shows a general increase at the top of the core from c. 900 cal year BP. Similarly, the largest size-class (> 63 µm) is characterised by extremely low levels at the bottom of the core, and only increases consistently from c. 900 cal year BP. The abundance of charcoal particles is relatively low (< 100 particles per slide) at the base of pollen zone Olp I before it increases to > 200 particles per slide towards the upper boundary of Olp I. Zone Olp II is marked by a sudden rise in charcoal to 300 particles at 255 cm, before dropping to low levels (< 100 particles) in this zone. Zone Olp III is characterised by a drastic change in charcoal abundance compared to any other zones. This zone is characterised by more sustained peaks rising to 2500 particles in the uppermost samples towards the top of the core.

## Discussion

### Palaeovegetation dynamics of the wider Laikipia Plateau from 2268 cal year BP to c. 1615 cal year BP

Sedimentary data from Marura Swamp provide a record of ecosystem history of the Laikipia Plateau dating from ca. 2268 cal year BP. By combining a range of proxies and bringing in context from archaeological sites, we can disentangle the roles that climate variability, taphonomic processes (Lamb et al. 2003), and anthropogenic activities associated with pastoralism and agriculture have had on shaping the composition and distribution of the current ecosystems and land use on the wider Laikipia Plateau landscape (Fig. 2).

There was more intact forest relative to the present day from around 2268 through 1615 cal year BP that was dominated by *Podocarpus* among a mix of other montane forest taxa. Although *Podocarpus* is a taxa that produces large amounts of well-dispersed pollen, its representativity in sediments does reflect the abundance in ecosystems surrounding the core site (Marchant and Taylor 2000). The fungal spore data also indicate the Marura catchment was heavily forested before 1615 cal year BP as indicated by the presence of *Trichoglossum* cf. *hirsutum* and *Glomus* spores derived from soft-rot of wood (van Geel and Anderson; 1988). The Laikipia Plateau forms a corridor of low-lying rangeland between Mt Kenya (Fig. 4) and the Nyandaru Mountains where montane forest is currently present (Beentje 1994). Over the past 2000 years these mountains, like many across East Africa, have been a focus for human settlement, partly due to their fertile soils and reliable rains that help create a range of growing conditions. These settlements have typically resulted in the clearing of montane forest, as documented across East African mountains (Heckmann et al. 2014; Finch et al. 2017; Marchant et al. 2018). The period around c. 2300 cal year BP to present generally coincides with archaeological evidence indicating that the Laikipia Plateau was already a site of food production involving domesticated animals (Taylor et al. 2005; Lane, 2011). Major forest clearance appears to have occurred widely along the western part of Mount Kenya (Rucina et al. 2009), although this was not unidirectional and there are documented examples of subsequent phases of forest regeneration, likely in response to periods of higher rainfall although with differences in associated species compositions, as recorded in sediment cores from Lake Bogoria (van der Plas et al. 2019).

An expansion of farming communities after c. 2000 cal year BP most probably accounts for initiating the clearance of large expanses of montane forest for agriculture (Lamb et al. 2003). This impact on the surrounding mountains is in contrast to the rangelands over the same period (c. 2150–1500 cal year BP) where the regional pollen sequence records a period of limited human impact on the Laikipia Plateau (Taylor et al. 2005). People were certainly a common part of the rangeland landscape, and the available archaeological information indicates the presence of domestic livestock as early as c. 4100 cal year BP (Lane 2011). Thus, it is likely that differential ecosystem responses to early human settlement reflect the nature of the land use and associated transformation. Populations settling on mountain environments would have had to clear the land for agricultural activity, or at least develop agroforestry systems characterised by selective forest clearance (Fernandes et al. 1984). Conversely, the spread of pastoralism entailed the addition of grazing animals into a system in which wild grazers were an integral, dominant component, and so worked within the existing rangeland ecosystem. This is not to say there were no ecological changes arising from the introduction of pastoralism. Vegetation management, for example, would have reduced the threat posed by various disease vectors (Gifford-Gonzalez 1998). The replacement of Afromontane forest from c. 1615 cal year BP may even signal wider forest clearance associated with the expansion of agriculture *and* pastoralism (Taylor et al. 2005), as landscapes were steadily ‘domesticated’. Ongoing process of climate change operated concurrently with this increase in human–ecosystem interaction. As apparent from other high-resolution palaeoenvironmental studies from nearby Lake Naivasha (Lamb et al. 2003), Mount Kenya (Rucina et al. 2009), and southern Kenya (Githumbi et al. 2018), for example, there were reduced levels of effective precipitation around 2200 cal year BP with enhanced seasonality likely reflected by pronounced low stands in the levels of Lake Tanganyika (Alin and Cohen 2003) and Lake Edward (Russell et al. 2003). These reductions in moisture regimes would have made the Laikipia Plateau less suitable for year-round occupation encouraging a shift towards lowland–highland seasonal migration between the Rift Valley and Laikipia, with reduced opportunities for more sedentary livelihoods, such as those based on crop cultivation.

### 1582 cal year BP to present

From 1582 cal year BP, montane forest continued to decline with increased abundance of Poaceae and secondary forest species that included *Croton*, and to a lesser extent *Rapanea*, that indicate a more open vegetation type. The presence of *Phyllanthus,* which is commonly associated with cleared forests, is further indication that montane forest reduction was the likely result of anthropogenic activities. Evidence of human disturbance is additionally suggested by increases in the amounts of *Justicia* and Asteraceae, which are often associated with human activities (Lind and Tallantire 1971). Against this backdrop of reduced regional montane forest cover and increasingly dominant Poaceae (produced by a wide range of grasses such as *Panicum maximum*, *Pennisetum purpureum*, and *Setaria sphacelata*), local riverine moist evergreen forests persisted as documented by *Cyathea*, ferns such as *Pteris* and fungal spores (*Sordaria*-type) indicative of edaphically wet conditions close to the coring site. The record of swamp forest growth is a good indication that Marura Swamp was functioning well ecologically, able to retain sediments and nutrients. Today, swamps like Marura have crucially important functions in the extensive savanna landscape as they provide a locus of dry season grazing for wildlife and domestic stock (Fig. 4), and can be a vital refuge during episodic droughts, which are becoming more frequent across East Africa. For example, during extensive droughts the wetlands inside and outside the Amboseli National Park in southern Kenya act as a lifeline to the ungulate populations, with the consequence that although the numbers of large herd populations can be decimated, they are not locally extirpated (Western and Manzolillio-Nightingale 2003).

The increased presence of dung fungi ascospores after 1582 cal year BP suggests that herbivores were also present at the site; *Ustulina*, for instance, is a particularly good indicator of domestic animals (van Geel 2001). *Valsaria* spores are also associated with herbivore dung (Davis 1987), and are common on the dung of cattle, sheep, and goats. However, one of the challenges of interpreting spore signatures, particularly in the context of East Africa where there are extensive herds of wildlife, is that these also grow on the dung of wild herbivores (Ahmed and Cain 1972). Indeed, the fungal spores from herbivore dung in this record are likely to have been produced by both wild herbivores and domestic livestock with changing proportions reflecting the overall amount of dung in the landscape. For example, the marked increase in *Cercophora* spores during the period of forest decline after c. 1615 cal year BP suggests an increase in the concentration of herbivores in the area, while their overall increase over the last 1500 years probably reflects an increased presence of domesticated stock. This proposed shift in the type of herbivores on the Laikipia Plateau is also supported by the available archaeological data which indicate an early presence of domestic livestock on Laikipia by c. 4100 cal year BP (at Ol Ngoroi in the Lolldaiga Hills), and a steady expansion of pastoralism numbers from c. 3000 cal year BP and especially after ca. 1200 cal year BP, as reflected by the increase in the number of archaeological sites (Fig. 2) with the remains of domestic livestock and artefact types diagnostic of the Pastoral Neolithic associated with the initial phases of expansion, and the subsequent Pastoral Iron Age (Siiriäinen 1984; Causey 2010; Lane 2011). Based on the density of Pastoral Iron Age sites and the size of some of them, including at Mugie and Maili Sita (Fig. 2), by c. 500–450 cal year BP pastoralist communities were well established across much of the Laikipia Plateau and the adjacent Leroghi Plateau, interspersed with clusters of hunter-gathering communities, as in the Mukogodo Hills (Mutundu 1999).

One of the key and pervasive environmental shifts supported by the charcoal, pollen, and fungal spore evidence is a sudden and sustained increase in fire activity on the Laikipia Plateau from around 1720 cal year BP towards the top of the core. As fires, fungal spores, herbaceous taxa, and Poaceae increased, other Afromontane vegetation continually decreased. Increased burning of vegetation on the Laikipia Plateau may be linked to regional clearing of Afromontane forest on the adjacent highlands as sedentary agriculture continued to expand (Marchant et al. 2018). More locally on the Laikipia Plateau, fires may have been used to improve and extend grazing land, possibly also reducing the threat of insect-borne diseases such as *Trypanosomiasis* (Gifford-Gonzalez 1998), and perhaps even clearing land for localised crop cultivation during periods of increased rainfall.

Increased fire activity, as recorded by relatively abundant charcoal, is particularly evident from around 880 cal year BP to present; the greater abundance of large sized charcoal particles could indicate fires had a more local origin. According to Clark and Royall (1995) and Marlon et al. (2016), charcoal particles larger than 63 μm reflect local burning, as smaller particles can be transported longer distances and often derive from regional and extra-regional fires. From a human settlement perspective, the archaeological record indicates this period spans the terminal stages of the Pastoral Neolithic and the early part of the Pastoral Iron Age in the region, marked by an uptake in the use of iron implements and associated iron smelting activities (which would have contributed to the charcoal record) and potentially a growing importance of cereals in pastoralist diets and food production strategies (Lane 2013).

The onset of Pastoral Iron Age settlement in the area may have begun by ca. 850 cal year BP (Siiriäinen 1984), at which time the hunter-gatherer populations were apparently already occupying, or were displaced to, the upland hilly areas. Some clear archaeological evidence for these trends comes from the site of Deloraine (Ambrose et al. 1984) in the Rift Valley c. 200 km to the south. Traces of iron smelting activity have also been recorded at various locations across the Laikipia Plateau and the adjacent Leroghi Plateau, although dates from those sites that have been excavated are slightly later, clustering around the last three to four centuries (Iles and Martinón-Torres 2009; Iles and Lane 2015). Older examples of iron smelting, nonetheless, may await discovery through targeted archaeological fieldwork.

Additionally, high-resolution data from Lake Bogoria suggest crop cultivation (supplementing longer established herding) in the adjacent Rift Valley lowlands at least by c. 450 cal year BP (van der Plas et al. 2019), while a record from nearby Lake Baringo indicates land degradation arising from human activities by c. 300 cal year BP, which has persisted into the present (Kiage and Liu 2009). Instances of crop cultivation have yet to be confirmed on the Laikipia Plateau by archaeobotanical research, although unpublished phytolith data from the site of Maili Sita in the Lolldaiga Hills, dated c. 350–250 cal year BP, hint at such a possibility (VM pers. obs.). Such a shift is also supported by an increase in large Poaceae pollen grains in the record that could reflect an increase in cereal cultivation (Hamilton 1982), although this size-to-cereal relationship remains controversial (van der Plas et al. 2020). More sustained, systematic archaeobotanical sampling of multiple sites, especially those associated with Pastoral Iron Age ceramics, is clearly needed to test this hypothesis. Further evidence of direct human interaction with the wider environment that can also be used as additional time markers is derived from the consistent presence of exotic pollen taxa (*Eucalyptus* and *Pinus*) in the uppermost samples. *Eucalyptus*-type pollen may be derived from the exotic timber tree *Eucalyptus*; however, this pollen type could also be derived from *Eugenia* or *Syzygium* which produce similar morphotypes (Finch et al. 2017) and could explain its present at spot samples lower down the upper part of the sediment profile. *Pinus* is a more diagnostic pollen type and although there are no exotic plantations on the Laikipia Plateau there are plantations on the nearby slopes of Mount Kenya and the Mau Highlands that have been present since the early twentieth century.

#### Relevance of past pastoral rangeland interactions to addressing contemporary challenges

East African pastoralists move their livestock in response to rainfall and grazing regime in order to maximise herd size, and milk and meat yields (Western and Finch 1986). Herding strategies, such as those of the Maasai and Samburu, are adapted to arid savannas, including selection and cross-breeding of livestock for drought tolerance (Kohler-Rollefson and McCorkle 2004), with the result that pastoral livestock breeds, including the zebu cattle of the Maasai, are extremely efficient at producing milk and meat under a highly seasonal climate (Coughenour et al. 1985). Communities will also select settlement sites according to the availability of water, forage, fencing, and fuelwood in a manner that allows them to minimise the impacts of climatic extremes, flooding hazards, disease, and predation (Western and Dunne 1979; de Leeuw and Wilson 1987). Land adjacent to Marura Swamp where water is available year-round and there is plenty of material for firewood, fencing, and house construction would have provided such a location.

As previous research has highlighted, precolonial pastoralist livelihoods revolved around optimising livestock forage intake by selecting the best grazing pastures in any season and minimising stock losses to drought, disease, and predation, including raiding (Homewood and Rodgers 1987). However, with a steady trend towards enclosure since the early twentieth century, coupled with land expropriation during the colonial period (Hughes 2006), population growth, and increased sedentism, the options open to pastoralists have narrowed and risks intensified as recent droughts have become more frequent and more intense. Across East Africa rangeland development has been held in check by livestock diseases and the local nature of meat markets. However, local demands for milk and meat are increasing, and livestock diseases can be controlled, with both intensifying pressure on the resource. Moreover, these pressures are most severe in arid and semi-arid areas where recent droughts have seen social disruption and mass emigration to frontier towns, marginal agricultural areas, forest reserves, conservancies, and National Parks. Today, for example, the Laikipia Plateau comprises a mosaic of high-profile conservation initiatives and mixed commercial and subsistence pastoralism (Fig. 2), all with competing and overlapping land uses and demands (Boles et al. 2019). Within this framework, there is growing pressure on the Laikipia Plateau rangelands to provide high, sustained yield of milk and beef at economic cost while at the same time there have been moves to allocate some of these rangelands to solar energy production (Bersaglio 2017). Projected population increases will likely result in more land being used for settlements, further reducing rangeland area and increasing human–animal conflicts.

In attempting to resolve these kinds of conflicts, conservation and rangeland policies often lack important information pertaining to long-term environmental change and patterns of land use (Brockington and Homewood 2001; Gillson and Marchant 2014). Adequate knowledge is needed for policy makers to implement effective land use management schemes to protect both wildlife and the ecological patterns that sustain them (Boles et al. 2019). At the same time, local concerns regarding ancestral rights to land, natural resources, and other kinds of common property must also be addressed. In particular, conservation and land use policies and actions should dispense with myths that marginalise pastoralists such as that pastoralism creates deserts, that there are more cattle than ever, that sedentary commercial ranching is superior to mobile pastoralism, and that pastoralists will recover and resume life as normal after extended droughts (Western and Manzolillio-Nightingale 2003). Instead, policies and actions should be grounded in realities and longer-term perspectives, such as that presented here from sedimentary archives and archaeological data, that can provide grounded realities on the impacts of ecosystem change and human interaction.

## Conclusions

This study shows clearly that ecosystems on the Laikipia Plateau and adjacent highland areas are highly variable and respond to a series of climate and anthropogenic pressures. It further highlights that the regional vegetation, especially the relative abundances of grassland and woody species of the savanna and Afromontane biomes, has been incredibly dynamic. Human impacts have also played an increasing role over the last c. 2268 years as pastoralism spread through the region. Grazing and browsing by domestic stock, wildlife, the use of fire for disease vector control and rangeland management, and clearance of woodland and forest for the extension of both pasture and arable land have all had an impact on the composition of the vegetation. In particular, the presence of fungal spores and charcoal evidence an opening of the forest ecosystem and suggest the increasing presence of pastoralists at least from c. 1615 cal year BP to the present.

The initiation of a regular cycle of pastoralist settlement formation, occupation, and abandonment would have led to the generation of distinctive nutrient hotspots across these savanna grasslands (Boles and Lane 2016). Such abandoned former pastoralist settlements contribute to the formation of distinctive grazing lawns (sometimes referred to as ‘glades’) that can be sustained for centuries, perhaps even millennia, through a suite of relations of ecological mutualism at multiple trophic levels. Intensification of a human footprint on this landscape also likely stimulated other transformations in land cover such as *Acacia* replacing Afromontane taxa as fire intensity increased. Cereals (Poaceae > 60 µm) and ruderal taxa reflecting settlement increased after c. 800 cal year BP, consistent with archaeological data suggesting increased human activity on the Laikipia Plateau associated with the Pastoral Iron Age. The archaeological record provides evidence of shifts in settlement and mobility, while other patterns of human distribution indicate that these pre-industrial populations were susceptible to climatic changes prompting a variety of adaptive responses. Later colonial administration expanded to these areas introducing exotics to replace the cleared Afromontane forests around 1915, shortly after forced removal of most of the remaining pastoralist populations.

In summary, this combination of different environmental proxies from a sedimentary archive on the Laikipia Plateau, interpreted against the context of a growing body of archaeological data, provides a rich insight into the vegetation history of the area in a manner that can help contextualise current debates around conservation, rangeland management, and ecosystem response to climate change and human interaction.

## Data Availability

All data are available in the main text.
